# Robust muscle force prediction using NMFSEMD denoising and FOS identification

**DOI:** 10.1371/journal.pone.0272118

**Published:** 2022-08-03

**Authors:** Yuan Wang, Fan Li, Haoting Liu, Zhiqiang Zhang, Duming Wang, Shanguang Chen, Chunhui Wang, Jinhui Lan

**Affiliations:** 1 Beijing Engineering Research Center of Industrial Spectrum Imaging, School of Automation and Electrical Engineering, University of Science and Technology Beijing, Beijing, China; 2 National Key Laboratory of Human Factors Engineering, China Astronaut Research and Training Center, Beijing, China; 3 School of Electronic and Electrical Engineering, School of Mechanical Engineering, University of Leeds, Leeds, United Kingdom; 4 Department of Psychology, Zhejiang Sci-Tech University, Hangzhou, China; Universita degli Studi di Milano, ITALY

## Abstract

In this paper, an aliasing noise restraint technique and a system identification-based surface electromyography (sEMG)-force prediction model are proposed to realize a type of robust sEMG and muscle force prediction. For signal denoising, a novel non-negative matrix factorization screening empirical mode decomposition (NMFSEMD) and a fast orthogonal search (FOS)-based muscle force prediction model are developed. First, the NMFSEMD model is used to screen the empirical mode decomposition (EMD) results into the noisy intrinsic mode functions (IMF). Then, the noise matrix is computed using IMF translation and superposition, and the matrix is used as the input of NMF to obtain the denoised IMF. Furthermore, the reconstruction outcome of the NMFSEMD method can be used to estimate the denoised sEMG. Finally, a new sEMG muscle force prediction model, which considers a kind of candidate function in derivative form, is constructed, and a data-training-based linear weighted model is obtained. Extensive experimental results validate the suggested method’s correction: after the NMFSEMD denoising of raw sEMG signal, the signal-noise ratio (SNR) can be improved by about 15.0 dB, and the energy percentage (EP) can be greater than 90.0%. Comparing with the muscle force prediction models using the traditional pretreatment and LSSVM, and the NMFSEMD plus LSSVM-based method, the mean square error (MSE) of our approach can be reduced by at least 1.2%.

## 1 Introduction

When the astronauts work in the microgravity environment of space stations for an extended period, they experience bone loss and muscle atrophy [1–3], resulting in a substantial loss of muscle strength, which impacts the astronaut’s physical health and the ability to perform tasks, such as handling equipment and opening cabin doors. After returning to Earth, astronauts typically need a period of rehabilitation training to regain their physical health. Direct and indirect measurement methods are commonly used to assess muscle strength. The former approach causes some harms to the human body and is not portable when astronauts are active, so it has limited application. The latter method evaluates muscle force by analyzing the surface electromyogram (sEMG) signals of an astronaut. Clearly, this technique is an efficient and noninvasive technique with a broad application. Research on muscle force prediction is useful for monitoring the muscle force status of astronauts in real-time and developing targeted on-orbit rehabilitation training programs.

The muscle force prediction model, including the physiological and the phenomenal models, establishes a nonlinear relationship between the sEMG signals and muscle force. A typical physiological model, the Hill model, is based on various physiological parameters during muscle contraction [4]. Its simplified model has good adaptability and low computational amount because it ignores muscle fatigue; however, its accuracy is poor and can only predict one muscle. Researchers have committed to finding an optimal strategy with low complexity and high accuracy by using sensitivity analysis [5, 6]. The phenomenal model includes polynomial fitting, artificial neural networks, and system identification. When using polynomial fitting, some authors predicted the joint torque of biceps brachii [7]; other authors found that muscle force prediction was the best when the polynomial fitting order was more than three [8]. The polynomial fitting has a simple structure and shorter training time; however, it has low accuracy. Thus, some researchers adopted a neural network to predict hand and palm-grip forces during elbow motion [9, 10]. The accuracy of an artificial neural network depends on its structure and parameters, and the training typically takes a long time.

Another phenomenal model used for muscle-force prediction is the system identification method. Popular methods are parallel cascade identification (PCI) and fast orthogonal search (FOS). Some authors used PCI to build a relationship between the upper arm sEMG signals and wrist induction force [11]. PCI considered the dynamic linear finite impulse response and static nonlinear fitting, which realized the fusion of dynamic and static information. However, it took a long training time. Conversely, the FOS method established prediction models by minimizing the mean square error (MSE) between the estimated and true values of the system output [12]. Some authors used FOS to predict the muscle force of upper arm and forearm muscles during elbow flexion and extension [13]. Thus, FOS quickly achieved muscle force prediction and resulted in high accuracy by building suitable candidate functions.

Surface EMG signals have non-linear and non-stationary characteristics, and are usually processed by time-frequency analysis. However, traditional analysis is based on Fourier analysis, which cannot express local frequency change with time. Empirical mode decomposition (EMD) overcomes this limitation and is widely used in physiological signal analysis, fault diagnosis, and other fields. EMD has problems such as modal aliasing and end effect [14]. Scholars have proposed improved EMD methods to suppress modal aliasing. For example, research on improvement based on noise assistance, ensemble empirical mode decomposition (EEMD), and noise assisted multivariate empirical mode decomposition (NAMEMD) were realized by adding white noise to EMD decomposition [15–17]. Research on improvement based on mathematics, including local fitting curve replaced cubic interpolation curve [18]. Research on improvement based on EMD decomposition characteristics, independent component analysis (ICA) was used to eliminate mode aliasing in EMD decomposition results [19].

In this study, we propose a non-negative matrix factorization screening empirical mode (NMFSEMD) denoising approach for removing the aliasing noise in the sEMG signals by analyzing the noise distribution of sEMG at various time characteristic scales. Using the denoised sEMG signal as the input of muscle force prediction model, the reliability and accuracy of the model can be improved. Additionally, we apply the system identification model FOS to establish the prediction model. We preset several forms of candidate functions according to the time-domain characteristics of sEMG signals, then employ an iterative optimization technique to select several basis functions, and use their weighted sums as the muscle force prediction model.

The major contributions of this investigation are as follows: a novel NMFSEMD method for denoising sEMG signals is proposed and a candidate function in the form of a derivative to the FOS model to predict the muscle force is considered. This noise reduction technique has three main advantages: (i) as an improved algorithm of EMD, NMFSEMD can better suppress mode mixture and improve the data reliability of sEMG in muscle force prediction; (ii) NMFSEMD can distinguish the noisy intrinsic mode functions (IMF) components and the real intrinsic mode functions components (IMFs) from the EMD decomposition results by using the designed IMFs screening process; (iii) NMFSEMD is a data fusion method essentially and can extract several sequences that can best represent the state of muscle activation when used in sEMG signals.

The remainder of this paper is organized as follows. First, we will introduce the principle of proposed NMFSEMD. Second, we present the process of building a muscle force prediction model using FOS. Finally, experimental findings and discussions will be presented.

## 2 Proposed sEMG-force-prediction method

**Fig 1:**
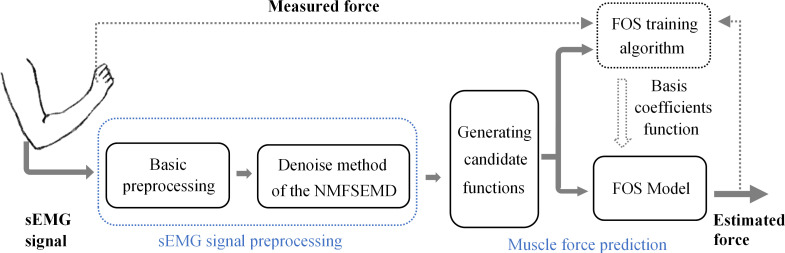
Computational flowchart of NMFSEMD denoising and FOS-based muscle force prediction using the denoised sEMG signals.

In the muscle force prediction process, the sEMG signal preprocessing is first considered to improve the reliability of modeling data. Conventional preprocessing methods are initially used for sEMG signal, such as outlier detection. Then, the denoising and preprocessing of sEMG signals are conducted by integrating the EMD with NMF, i.e., the proposed NMFSEMD denoising method. Subsequently, the denoised sEMG signals are obtained and used as the input of the muscle force prediction model. Then, basic functions of FOS are computed using the denoised sEMG signals in the form of preseted candidate functions. Finally, the muscle force prediction model represented by the linear combination of several basic functions is obtained and can be calculated through FOS model training by comparing the measured and predictive muscle forces in the model training process. The flowchart of sEMG-based muscle force prediction is shown in Fig 1.

### 2.1 sEMG signal denoising using EMD and NMF

#### 2.1.1 EMD method

While acquiring the sEMG signals, the power line interference, ambient noise, motion artifact interference [20–22], and other noises can be observed. The frequency range of sEMG signals is 0.0 to 500.0 Hz [23], and the main frequency range of sEMG signals is 50.0 to 150.0 Hz. The frequencies of motion artifact interference, ambient noise, and power noise are from 0 to 20.0 Hz, 40.0 to 60.0 Hz, and 50.0 Hz, respectively. The traditional noise reduction methods used for analyzing the sEMG signals are bandpass filtering, wavelet filtering, EMD, or blind source separation (BSS) [21, 24–26]. However, the bandpass filtering method cannot filter out the noise mixed in the main spectrum. Moreover, it is different to use the wavelet filtering method to accurately evaluate the denoising results. The BSS method has many strict restrictions and requirements. For example, the source signals obey Gaussian distribution and their intensities should be less than 1.0; each source signal is statistically independent. According to its characteristic time scale, the EMD approach can adaptively decompose the signal into a series of IMF with frequency values ranging from high to low. The characteristic time scale denotes the signal change process that can be directly observed from the time-domain and represent the local frequency features. Thus, the noise in sEMG signals can be removed by processing intrinsic mode functions components (IMFs) which containing different frequency information.

The theory of EMD is based on the idea that any real signals can be decomposed into a set of simple IMFs, which are independent of each other [27]. EMD decomposes the fluctuations of different scales in a signal step by step and produces a series of data sequences of different scales. Each sequence is called an IMF. Each IMF should satisfy two conditions [28–30], namely, the difference between the number of local extreme points and zero points is not more than one; and at any time, the average value of upper envelope formed by the local maximum point and the lower envelope formed by the local minimum point is zero. The calculation process of EMD is as follows:

1. The upper and lower envelopes are obtained by cubic spline interpolation of the local maxima and local minima points of signal *x*(*t*), respectively.
2. The difference *h*(*t*) between *x*(*t*) and the average value *m*(*t*) of the upper and lower envelopes is calculated.
3. If *h*(*t*) meets both the IMF conditions, it is the first IMF; otherwise, it is the new *x*(*t*). The preceding steps are repeated until the difference after *k* times satisfies the IMF conditions and is recorded *c*(*t*) = *h*_*k*_(*t*) as the first IMF.
4. The existing IMF *c*(*t*) from *x*(*t*) is removed to obtain the remaining component *r*(*t*), and *r*(*t*) is considered as the new *x*(*t*). Then the preceding steps are repeated to get the remaining (*n–* 1) IMFs.
5. When the residual *r*_*N*_(*t*) is a monotone function, the algorithm can be stopped. The expression of signal *x*(*t*) after EMD decomposition is shown in Eq (1).

$$x(t)=\sum_{n=1}^{N}c_{n}(t)+r_{N}(t)$$ (1)

where *c*_*n*_(*t*) (*n* = 1, …, *N*) is the IMF and *r*_*N*_(*t*) is the residual error.

#### 2.1.2 NMF method

Some noises in the sEMG signals can be filtered out by discarding the corresponding IMFs after EMD decomposition [15]. However, the noises mixed in the main spectra of signals still remain. Thus, the BSS method is used to filter out these noises because it can recover “source signals” that cannot be directly observed from the “mixed signals” [31]. BSS methods include principal component analysis (PCA) [32], ICA [33], and NMF [34]. PCA uses the linear transformation of matrix to reduce data dimensions and eliminate redundant data. ICA and EMD decompose the matrix to find the basis vectors representing the local features of the observed matrix. ICA and EMD are more suitable than PCA for obtaining muscle activity feature from the sEMG signals. Additionally, ICA results must be statistically independent, with at least one component obeys Gaussian distribution, and the observed signal dimension should not be less than the decomposed dimension. However, the distribution and quantity of NMF results have no specific requirements, and the non-negative of the decomposed matrix makes the result more meaningful in physiological signal analysis. Thus, NMF is more suitable for denoising the sEMG signals in this work.

For a given non-negative matrix *V*, the basic principle of NMF can be described in [34]. Herein, we find two non-negative matrices *W* and *H*, whose product approaches *V*, as shown in Eq (2).

$$V\approx WH,\;\{V\in R^{m\times n},W\in R^{m\times r},H\in R^{r\times n}\}$$ (2)

where *r* is the target dimension, *m* is the dimension of input matrix row, and *n* is the dimension of input matrix column. The key of NMF is the construction of loss and optimization functions. The loss function based on Euclidean distance and Kullback-Leibler divergence can meet the requirements of matrix factorization in NMF [35]. First, we initialize *W* and *H*, and then their iterative equations are computed by minimizing the optimization function. The NMF algorithm converges in a finite number of iterations.

#### 2.1.3 Proposed NMFSEMD algorithm

**Fig 2:**
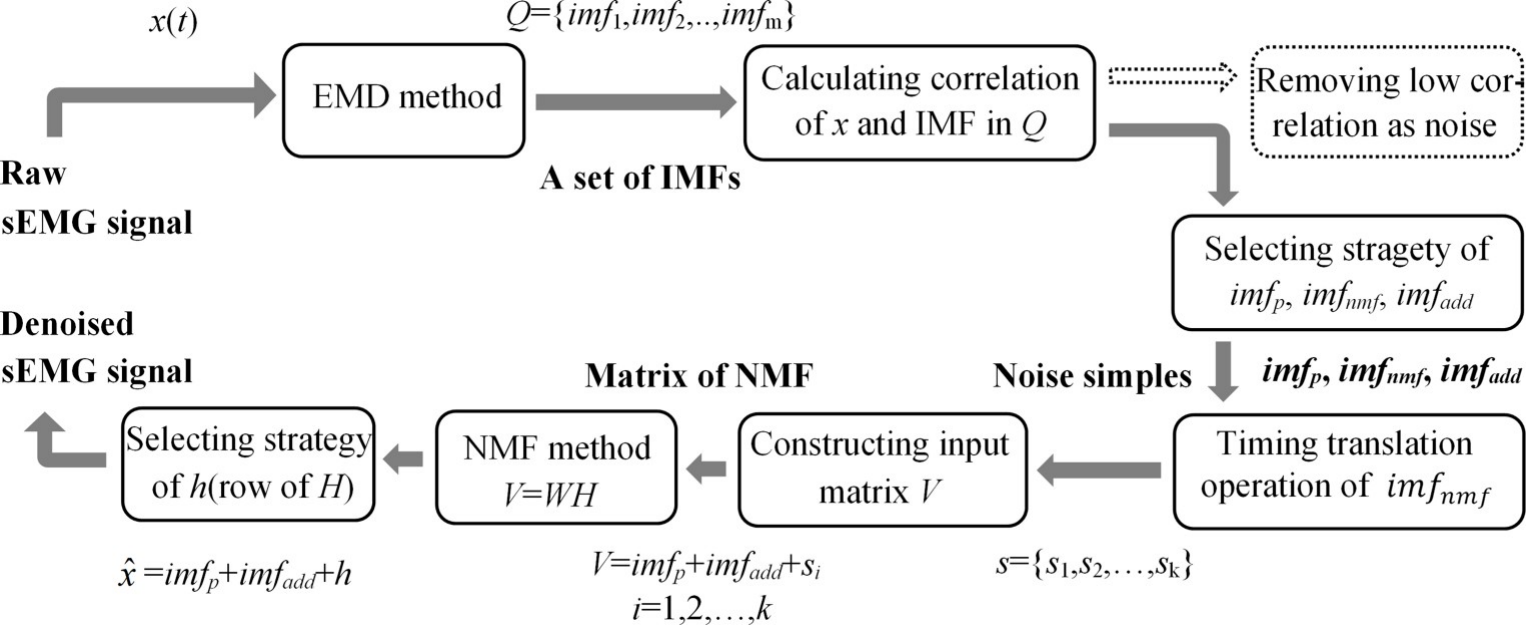
Concept flowchart of the proposed NMFSEMD method for denoising.

EMD can decompose the raw sEMG signals into a series of IMFs with different time scales; however, not every IMF contains useful information about the raw signal. To distinguish the IMFs with useful information and the IMFs with noisy information, we design their respective processes to achieve noise reduction in this section, i.e., an NMFSEMD denoising method is proposed. The implementation process of the NMFSEMD method is shown in Fig 2.

The designs of NMFSEMD method are as follows:

First, a set of IMFs with decaying frequencies can be obtained using the EMD. The frequency spectrum of each IMF is a part of the raw sEMG signal spectrum, whereas the total frequency distribution of IMFs is the frequency distribution of signal. For a set of IMFs, the higher the index of an IMF is, the higher its rank would be. The low-rank IMFs have a smaller time scale and a wider frequency domain, whereas the high-rank IMFs have a larger time scale and a narrow frequency domain. According to the spectrum analysis of sEMG noise presented in Section 2.1.1, the IMFs distributed in the low frequency mainly represent the low-frequency noise in the signal, and the noise can be eliminated directly by removing the corresponding IMFs. However, low-rank IMFs with a wider frequency domain overlap the main spectrum of sEMG. Thus, it is difficult to filter out these noises by removing IMFs. This unfavorable phenomenon where the noise frequency overlaps with the main frequency of signal is called the modal aliasing problem in EMD. That is, multiple time feature scales appear in one IMF, or one-time feature scale appears in multiple IMFs [26]. Ideally, each IMF only contains one time feature scale [27]. The proposed NMFSEMD method can address this modal aliasing problem by screening the IMFs, filtering out the noise in them, and finally reconstructing the denoised sEMG signal containing the main muscle activation information.

The specific steps of NMFSEMD are as follows:

1. The noisy sEMG signal is decomposed using the EMD method to obtain a set of IMFs with different time scales: *IMFs* = {*imf*_1_, *imf*_2_, …, *imf*_*m*_}.
2. In the abovementioned qualitative analysis of IMFs, the IMFs indicating noise should be removed, the IMFs with modal aliasing should be denoised, and the IMFs containing the main information of signal should be directly used for signal reconstruction. To achieve this goal in quantitative analysis, the concept of “correlation” is used to identify different types of IMFs. The detailed steps are as follows:

- First, the Pearson correlation value between each IMF and raw sEMG signal is calculated, as shown in Eq (3).
$$R(x,imf_{i})=\frac{\sum_{t=1}^{N}\left[x-\bar{x}][imf_{i}-{\bar{imf}}_{i}\right]}{\sqrt{\sum_{t=1}^{N}{\left[x-\bar{x}\right]}^{2}}\sqrt{\sum_{t=1}^{N}{\left[imf_{i}-{\bar{imf}}_{i}\right]}^{2}}}$$ (3)
  where *x* is the raw sEMG signal, *imf*_*i*_ is the *i*-th component of the IMF set, *n* is the number of sampling points, $\bar{x}$ is the mean value of *x*, and ${\bar{imf}}_{i}$ is the mean value of *imf*_*i*_. The value of *R* is in the range [−1, 1]. The higher the value of *R* is, the stronger the correlation between *x* and *imf*_*i*_ would be.
- Second, *imf*_*add*_, *imf*_*nmf*_, and *imf*_*p*_ are searched according to the obtained correlation set: $CR=\{R(x,imf_{1}),R(x,imf_{2}),\ldots ,R(x,imf_{m})\}$. The symbol *imf*_*add*_ is used to construct the denoised signal and the input matrix of NMF. The symbol *imf*_*nmf*_ involves the phenomenon of model aliasing and should be denoised and then used to construct the denoised signal. The symbol *imf*_*p*_ contains the main information about the signal and is directly used to construct the denoised signal. Clearly, the *imf*_*p*_ does not exist in all IMF set. The selection strategies of three IMFs are as follows: *cr*_*th*_ called the preseted correlation threshold, which is an empirical boundary value that determines whether the IMF treated as noise is to be discarded. The symbol *cr*_*e*_ is called the empirical maximum correlation, which is the maximum correlation at the positions where the higher correlations often appear in set.

**The selection strategy of IMFs.**

/* *Assumption* */

    Set the lower and upper correlation limits for searching *imf*_*add*_, *imf*_*nmf*_, and *imf*_*p*_:

        *lower = MIN* (*cr*_*th*_, *cr*_*e*_) and *upper = MAX* (*cr*_*th*_, *cr*_*e*_)

    The set *CR* can be divided into three parts based on limited value, the position index set of each part, and

    IMFs set of the corresponding position indexes are as follows:

        *ind*_*a*_ = *INDEX* {*CR* ≤ *lower*}, *ind*_*b*_ = *INDEX* {*lower* < *CR* < *upper*}, and *ind*_*c*_ = *INDEX* {*upper* ≤ *CR*}

        *imfs*_*a*_ *= IMF* {*ind*_*a*_}, *imfs*_*b*_ *= IMF* {*ind*_*b*_}, and *imfs*_*c*_ *= IMF* {*ind*_*c*_}

/* *Algorithm* */

    **if** set *ind*_*a*_ and *ind*_*b*_ are not empty:

        **if** set *ind*_*c*_ is not empty, **then** *imf*_*p*_ is the superposition of all IMFs in set *imfs*_*c*_.

        **if** set *ind*_*b*_ has more than 1 IMF component, **then** *imf*_*nmf*_ is the IMF with the smallest correlation in set *imfs*_*b*_,

                    *imf*_*add*_ is the superposition of other IMFs in the set *imfs*_*b*_ except *imf*_*nmf*_.

        **else** *imf*_*nmf*_ is the IMF with the largest correlation in set *imfs*_*a*_, *imf*_*add*_ is the IMF in set *imfs*_*b*_.

    **if** set *ind*_*a*_ and *ind*_*c*_ are not empty, set *ind*_*b*_ is empty:

        **if** set *ind*_*c*_ has more than 2 IMF components, **then** *imf*_*p*_ is the IMF with the largest correlation in set *imfs*_*c*_,

                                *imf*_*nmf*_ is the IMF with the smallest correlation in set *imfs*_*c*_,

            *imf*_*add*_ is the superposition of other IMFs in set *imfs*_*c*_ except *imf*_*p*_ and *imf*_*nmf*_.

        **else** *imf*_*p*_ is the superposition of all IMFs in set *imfs*_*c*_,

            *imf*_*nmf*_ is the IMF with the largest correlation in set *imfs*_*a*_.

        **if** set *ind*_*a*_ is not empty, set *ind*_*b*_ and *ind*_*c*_ are empty:

            *imf*_*nmf*_ is the IMF with the largest correlation in set *imfs*_*a*_,

            *imf*_*add*_ is the superposition of other IMFs in set *imfs*_*a*_ except *imf*_*nmf*_.

    **end**

- Then, the IMFs that are not selected to construct *imf*_*add*_, *imf*_*nmf*_, and *imf*_*p*_ are removed as noises. Next, NMF is used to denoise the *imf*_*nmf*_.

3. Because the dimension of input matrix *V* in NMF algorithm is greater than 1, and the selected *imf*_*nmf*_ is a vector. Thus, the method of “signal time sequence translation” is used to construct the input matrix *V* in NMF. The details of this construction are as follows:

- First, this method cyclically shifts the sequence *imf*_*nmf*_ to the left for *p* positions and splices the left overflow part to the right end of the sequence. This translation operation is repeated to obtain *k* different noise samples: *s* = {*s*_1_, *s*_2_, …, *s*_*k*_}.
- Second, this method superimposes *imf*_*p*_ and *imf*_*add*_ as *imf*_o_: *imf*_*o*_(*t*) = *imf*_*p*_(*t*) + *imf*_*add*_(*t*).
- Then, this method accumulates *s* and *imf*_*o*_ (a row vector), respectively, to construct a noisy matrix *V*, as shown in Eq (4), whose dimension is *k* × *n*, where *n* is the duration of the raw sEMG signal.
$$V=[\begin{array}{c} imf_{o}(t)+s_{1}(t)\\ imf_{o}(t)+s_{2}(t)\\ \ldots \\ imf_{o}(t)+s_{k}(t) \end{array}]$$ (4)
  The signal-to-noise ratio (SNR) of *V* is approximately equal to the raw sEMG signal. Thus, the matrix *V* retains the effective components of raw signal, and this cyclic shift operation only alters the noise shape of raw sEMG signal.

4. Next, the NMF method is used to decompose the matrix *V*_*k* × *n*_ into the weight matrix *W*_*k* × *r*_ and the activation coefficient matrix *H*_*r* × *n*_ is defined according to Eq (2), where *k* is the number of channels of signal and *r* is the number of activation modes of signal. The *i*-th column of *W* represents the contribution of each channel to the *i*-th activation mode. The *i*-th row of *H* describes the trend of the *i*-th activation mode status changing with time, which is called the muscle activation curve. The steps that obtain the denoised *imf*_*nmf*_ from matrix *V* are as follows:

- The intensity of each activation mode can be measured by calculating the area surrounded by the muscle activation curve and time axis. Because the sampling frequency of the sEMG signal is high enough (i.e., 3000.0 Hz), the activation intensity can be calculated using Eq (5).
$$Intensity=\sum_{i=1}^{n}H(i),0<i\leq r$$ (5)
- After setting different *r* values, the corresponding number of muscle activation curves is obtained. In Section 3.4.2, we compare the denoising effect under different values of *r* and conclude that the denoising effect is best when *r* is equal to 2. Two muscle activation curves, *h*_1_ and *h*_2_, can be obtained, and the denoised *imf*_*nmf*_ can be expressed as: *imf’*_*nmf*_ = (*h*_1_ + *h*_2_)/2.

5. Finally, our method adds *imf’*_*nmf*_ and *imf*_*o*_: *x’*(*t*) = *imf’*_*nmf*_(*t*) + *imf*_*o*_(*t*), where *x’*(*t*) is the denoised signal of raw sEMG signal after applying the NMFSEMD denoising method, which is used in the subsequent muscle force prediction of FOS method.

### 2.2 sEMG-force modeling using FOS

The FOS method uses a series of linear or nonlinear basis functions and coefficients to establish a model whose output quickly approaches the real value of system to minimize the error between the estimated and actual values [12]. In the algorithm, an implicit orthogonalization method is used to transform the candidate functions into a set of orthogonal candidate functions [36, 37] {*p*_*m*_}, and their corresponding coefficients are expressed by *a*_*m*_, as shown in Eq (6). The MSE of the system is defined in Eq (7).

$$y(t)=\sum_{m=0}^{M}a_{m}p_{m}(t)+e(t)=\hat{y}(t)+e(t)$$ (6)

$$MSE=\bar{e^{2}(t)}=\bar{{\left[y(t)-\hat{y}(t)\right]}^{2}}$$ (7)

where $\hat{y}$ is the estimated system output, *y* is the true value of system, *e* is the model error, and{*p*_*m*_} is a set of orthogonal candidate functions to be selected.

All candidate functions are traversed by calculating the MSE reduction *Q*_*m*_ of each candidate function, and selecting the function add to model and remove from function set, which is corresponding to the largest *Q*_*m*_. We select a function from the remaining candidate functions and repeat the above steps until the termination conditions of FOS algorithm are met. The commonly used termination conditions include the following: the number of functions in the model reaches a preseted value; the model error is small enough, or the remaining candidate functions cannot considerably reduce the model error. When the search is stopped, the coefficients of each selected function can be calculated to complete the FOS algorithm modeling.

## 3 Experiments and results

A series of tests and evaluation experiments were performed to assess the validity and effectiveness of the proposed models and methods. All the simulation programs were written in Python (Pycharm2020) on our PC (32.0 GB RAM, 3.8 GHz Intel (R) Core (TM), and i7-10700K CPU).

### 3.1 Experimental data preparation

In this study, we investigated the preprocessing and modeling of muscle force prediction, and used the experimental data from [38]. The details of data are as follows. In the simulated weightlessness environment of bed rest, the sEMG signals of the biceps brachii, triceps, and brachioradialis muscles and the force signal of handgrip were collected synchronously during the push and pull processes. The maximum voluntary contraction was 100.0%; the signal acquisition frequency was 3000.0 Hz.

### 3.2 Evaluation of sEMG denoising method

In this section, the proposed denoising algorithm NMFSEMD is experimentally verified. Based on the detailed principle of NMFSEMD designed in Section 2.1.3, the key steps of this algorithm are analyzed visually and quantitatively. The implementation process of NMFSEMD algorithm is shown in Fig 2, every key steps in the process are verified in turn: the setting of correlation thresholds *cr*_*e*_ and *cr*_*th*_, the screening of IMF components based on threshold, the construction of noisy matrix using the idea of signal time sequence translation, the decomposition of noisy matrix considering the NMF and activation mode parameters, and the reconstruction and fusion of signal. Finally, the denoised sEMG signal can be obtained. Compared with the distribution of IMFs in time-domain and frequency-domain before and after signal denoising, it is verified that NMFSEMD algorithm can suppress the modal aliasing better than the traditional EMD.

**Fig 3:**
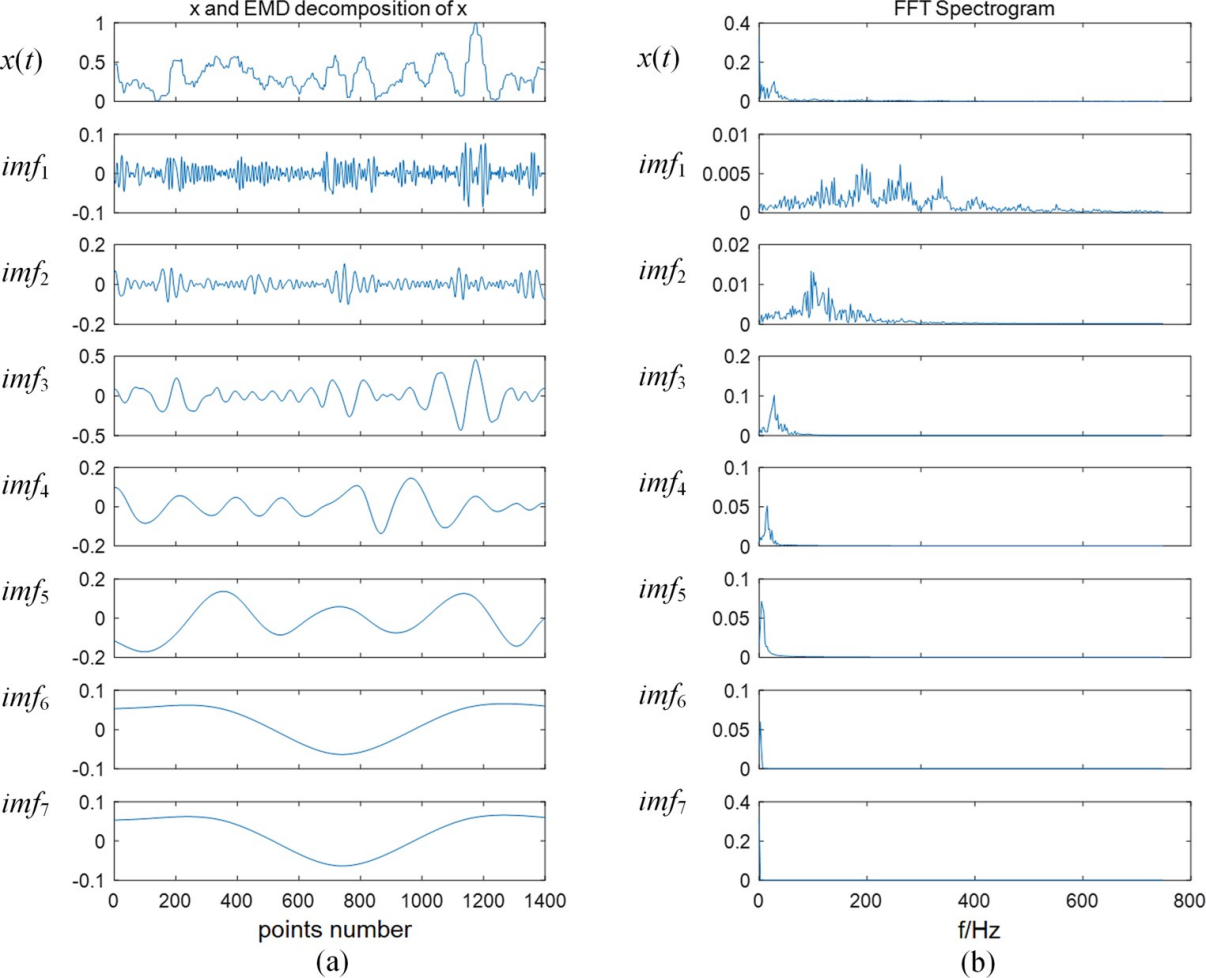
(a) Raw sEMG signal and its time-domain distribution diagram of a series of IMFs after EMD decomposition. (b) Frequency spectrum of raw sEMG signal and its series of IMFs’ frequency distribution diagrams obtained via decomposition after FFT computation.

The EMD method was used to decompose the sEMG signal *x*(*t*) and obtain the signal distribution on different time scales. The decomposed signal was denoted as *Q* = {*imf*_1_, *imf*_2_, …, *imf*_m_}. Fig 3(A) shows that the EMD decomposition result of biceps brachii signal has 1400.0 sampling points. Moreover, some IMFs, which are not periodic functions with a single time scale, can be observed, that is, a phenomenon of model aliasing is produced by noise. To more intuitively analyze noise in IMFs, the fast Fourier transform (FFT) is used to obtain the spectrum diagram of each IMF, as shown in Fig 3(B). The horizontal and the vertical axes denote the frequency and amplitude, respectively.

In the spectrum, the raw sEMG signal *x*(*t*) has a wide frequency domain and a large low frequency amplitude. An increase of the rank of IMFs makes the frequency distribution gradually approach the low frequency. The frequencies of high-rank IMFs are distributed in the range of 0.0 to 50.0 Hz, and no overlap is recorded in the main frequency of sEMG signals. In addition to the discussion in Section 2.1.3, *imf*_*add*_, *imf*_*nmf*_, and *imf*_*p*_ should be found to construct the denoised sEMG signal for clarifying the various types of IMFs. Thus, the results obtained on calculating the correlations between raw sEMG signal *x*(*t*) and its IMFs using Eq (3) are shown in Table 1.

**Table 1:** Pearson correlation between signals of the biceps brachii, triceps brachii, and brachioradialis muscles and their IMFs after EMD decomposition.

Coefficient	*imf*_1_	*imf*_2_	*imf*_3_	*imf*_4_	*imf*_5_	*imf*_6_	*imf*_7_
Biceps brachii	0.1019	0.1348	0.7658	0.2974	0.4914	0.1029	0.1064
Triceps brachii	0.0998	0.1267	0.4058	0.5794	0.4338	0.3575	0.2624
Brachioradialis	0.0553	0.1403	0.2916	0.5816	0.5215	0.4274	0.3870

The experimental analysis in this study is based on the sEMG signals of the biceps brachii. Results shows that the correlations of *imf*_7_ and other IMFs located near the edge of IMFs set are lower, while those of *imf*_3_ and other IMFs located near the middle of IMFs set are higher. According to Section 2.1.3, the selection strategy is used to determine *imf*_*add*_, *imf*_*nmf*_, and *imf*_*p*_. The empirical maximum correlation *cr*_*e*_ as 0.7658 can be obtained, and the preseted correlation threshold *cr*_*th*_ is set to be 0.35, according to Section 3.4.1. Thus, *imf*_*p*_ is *imf*_3_, *imf*_*add*_ is *imf*_5_, and *imf*_*nmf*_ is *imf*_4_.

Then, the noise mixed in *imf*_*nmf*_ should be filtered. First, the temporal shift of *imf*_*nmf*_ is used to obtain noise samples *s*. When the order of signal duration is 10^3^ and the order of magnitude of rows of *s* is about 10, the predicted result will have less error. Therefore, the dimension of *s* is set to 14 × 1400. Study in [39] has shown that when the sEMG signal duration was from 100.0 to 120.0 ms, it could exhibit some characteristics of an sEMG signal, therefore, the shift length of “signal time sequence translation” is set to 300 data points in this study. Then, by adding *s* and *imf*_*nmf*_, the input matrix *V* of NMF whose dimension is 14 × 1400 can be obtained. Subsequently, the matrix *V* is decomposed into the time-invariant activation mode matrix *W* and the time-varying activation coefficient matrix *H* using NMF. Further, it can be discovered that the activation information from signal is more reliable when the ranks of *W* and *H* are 2. The denoised *imf*_*nmf*_ can be obtained by calculating the mean of all rows of *H*. Therefore, the denoised signal *x’*(*t*) can be obtained by adding *imf*_*p*_, *imf*_*add*_, and the denoised *imf*_*nmf*_. The *SNR* and *EP* are used to evaluate the denoising effect, as shown in Eqs (8) and (9). And the *SNR* and *EP* results of sEMG signals after using NMFSEMD are shown in Table 2.

$$EP=\frac{\sum_{t=0}^{n-1}{\left|x'(t)\right|}^{2}}{\sum_{t=0}^{n-1}{\left|x(t)\right|}^{2}}$$ (8)

$$SNR=10\mathrm{lg}\frac{\frac{1}{n}\sum_{t=0}^{n-1}x^{2}(t)}{\frac{1}{n}\sum_{t=0}^{n-1}{\left[x(t)-x'(t)\right]}^{2}}$$ (9)

where *x* represents the raw sEMG signal, and *x’*(*t*) denotes the denoised signal. A high *SNR* value implies that the signal has a good noise reduction effect, whereas a high value of *EP* means the denoised signal can retain more characteristics of raw signal.

**Table 2:** Parameters of SNR and *EP* used for evaluating the denoising effect of biceps brachii, triceps brachii, and brachioradialis sEMG signals.

Raw sEMG signal	SNR/dB		EP/%
	Before denoising	After denoising	
Biceps brachii	0.00	13.55	92.12
Triceps brachii	0.00	17.37	99.23
Brachioradialis	0.00	16.46	94.87

**Fig 4:**
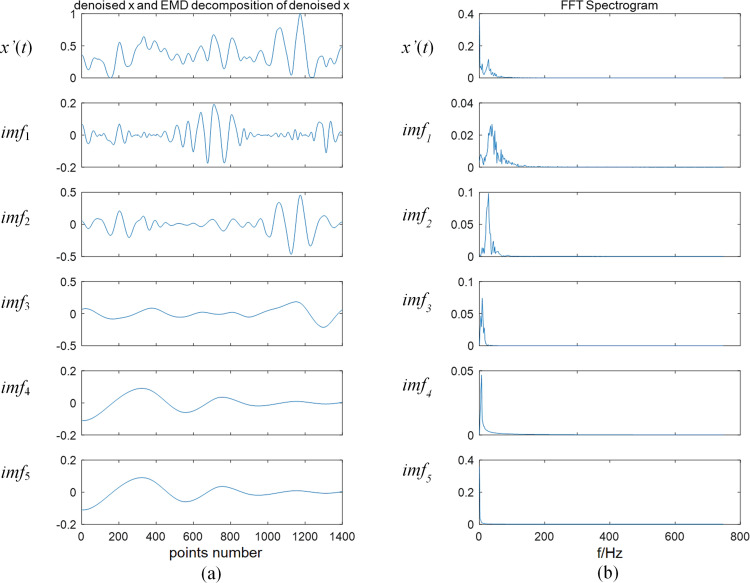
Results of denoising and signal processing. (a) Denoised biceps brachii signal *x’*(*t*) and its IMFs in the time-domain. (b) Corresponding frequency distribution of *x’*(*t*) and the IMFs after applying FFT method.

**Fig 5:**
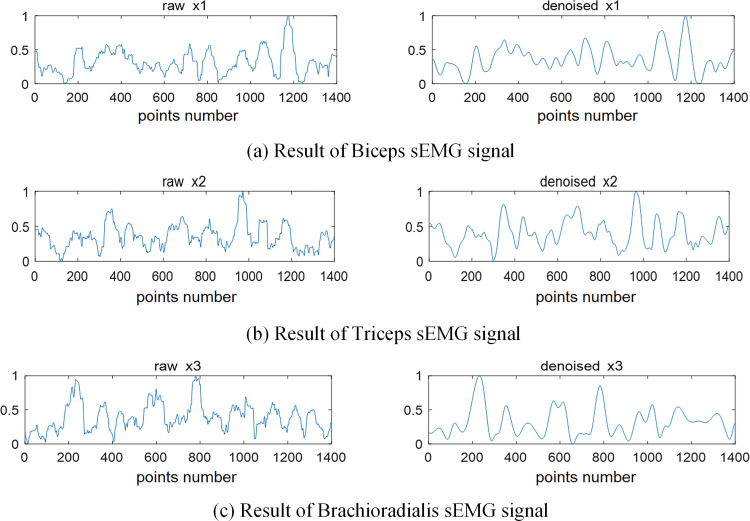
sEMG denoising results. The left side is the synchronous three channels raw sEMG signal, and the right side is the corresponding denoised sEMG signal using the proposed NMFSEMD. Three channels represent (a) biceps brachii signal, (b) triceps brachii signal, and (c) brachioradialis signal.

Based on the comparison of Figs 3 and 4, the frequency of denoised signal is mainly distributed in the middle frequency, and the low-frequency noise has been considerably reduced. The complexity of the time characteristic scale in IMF is reduced after the NMFSEMD denoising processing, and the spectrum curve of each IMF becomes smoother. Fig 5 shows that by comparing the sEMG signals before and after using the NMFSEMD denoising method, a smoother curve can be obtained after denoising with a better filtering effect.

### 3.3 Evaluation of the sEMG-force prediction model

The denoised sEMG signals obtained from the raw sEMG signals using the NMFSEMD denoising method are used as the input of muscle force prediction models. In this study, the FOS method is used to establish the muscle force prediction model. In this model, the candidate functions can be constructed using different function forms, as shown in Table 3. Because the raw sEMG signal used in this paper is the integrated electromyography value, which is added the candidate function in the form of a derivative. In Table 3, *E*_*bi*_, *E*_*tr*_, and *E*_*br*_ represent the denoised biceps brachii, triceps brachii, and brachioradialis signals, respectively. The expressions *sigm* and $\dot{x}$ represent the sigmoid function and first-order derivation of signal *x*, respectively.

**Table 3:** Set of candidate functions of FOS muscle force prediction model, which comprises the common, quadratic, square-root, sigmoid, and gradient function sets.

Common function	Square-root function	Sigmoid function	Gradient function	Quadratic function
*E*_*bi*_	$\sqrt{E_{bi}}$	*sigm*(*E*_*bi*_)	${\dot{E}}_{bi}$	*E*_*bi*_^2^
*E*_*tr*_	$\sqrt{E_{tr}}$	*sigm*(*E*_*tr*_)	${\dot{E}}_{tr}$	*E*_*tr*_^2^
*E*_*br*_	$\sqrt{E_{br}}$	*sigm*(*E*_*br*_)	${\dot{E}}_{br}$	*E*_*br*_^2^
*E*_*bi*_ × *E*_*tr*_	$\sqrt{E_{bi}\times E_{tr}}$	*sigm*(*E*_*bi*_) × *sigm*(*E*_*tr*_)	${\dot{E}}_{bi}\times {\dot{E}}_{tr}$	/
*E*_*bi*_ × *E*_*br*_	$\sqrt{E_{bi}\times E_{br}}$	*sigm*(*E*_*bi*_) × *sigm*(*E*_*br*_)	${\dot{E}}_{bi}\times {\dot{E}}_{br}$	/
*E*_*tr*_ × *E*_*br*_	$\sqrt{E_{tr}\times E_{br}}$	*sigm*(*E*_*tr*_) × *sigm*(*E*_*br*_)	${\dot{E}}_{tr}\times {\dot{E}}_{br}$	/

**Fig 6:**
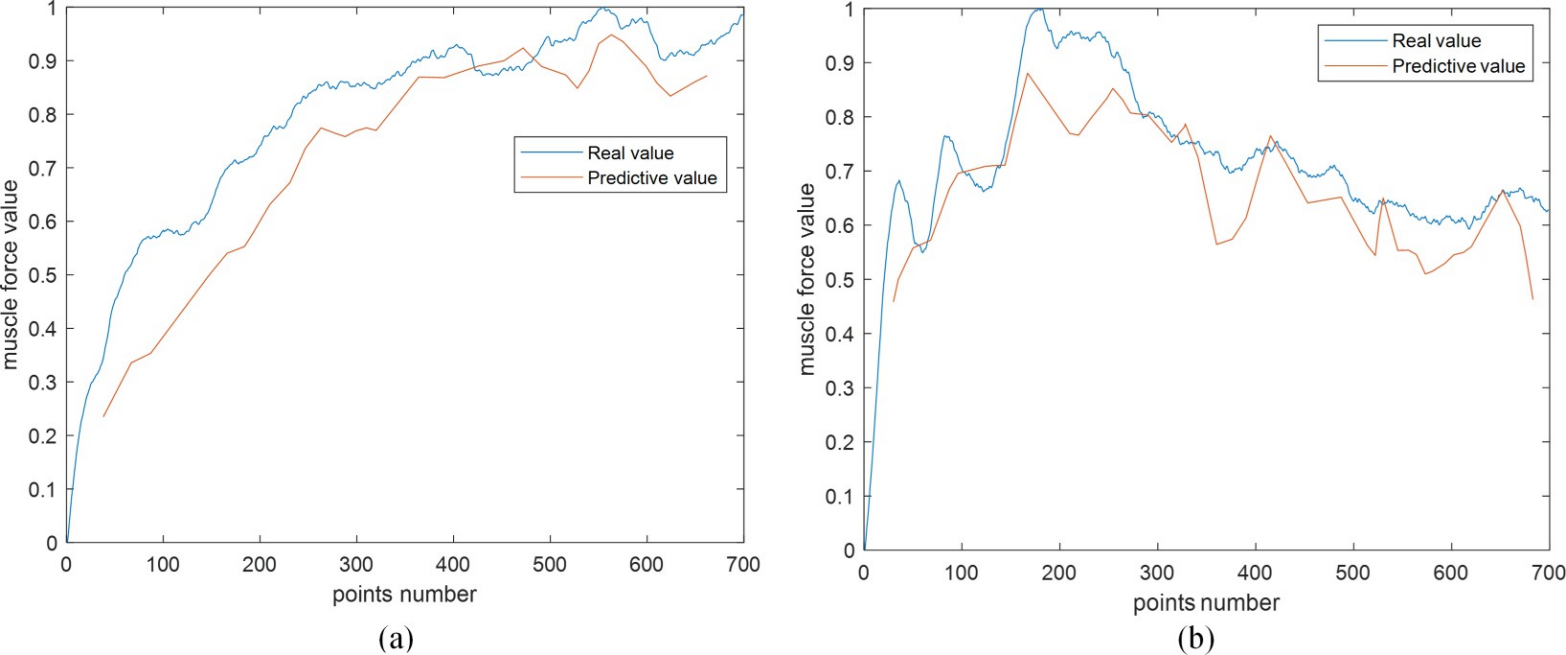
Comparison curves of the real and predictive muscle forces. (a) is the prediction result of experiment data1 in Table 4; (b) presents the results of experiment data2 in Table 4.

The denoised signals are used to construct candidate functions. However, the sEMG signal is weak, complex, and changeable, the waveform of candidate function varies considerably. Therefore, the predicted force outcome usually has a large oscillation. To better observe the prediction results, the direct predicted force is interpolated, and the average value of the upper and lower envelopes is taken to resist the fluctuation. Fig 6 shows the comparison curve between the real muscle forces and estimated results of FOS models.

Many experimental results verified the effect of applying the NMFSEMD method in the muscle force prediction model and the prediction effect of FOS prediction model. Table 4 shows when the FOS is used as the prediction model, the prediction results of input data that are not processed using the NMFSEMD method are compared with the input data that are processed using the NMFSEMD method. Additionally, Table 4 shows when the NMFSEMD denoising method is used as the preprocessing method, the results of prediction model that using the FOS method are compared with the model that using the LSSVM method. The prediction result is evaluated using the MSE value, as shown in Eq (7).

**Table 4:** MSE and its statistical error (mean ± standard error) of prediction results under different prediction models and input data in various experiments.

Result of MSE		Preprocessing method + prediction method		
		NMFSEMD + LSSVM	Traditional + FOS	NMFSEMD + FOS
**Experiment data1**	1	229.0534	505.3136	226.4261
	2	228.7566	505.4572	226.2916
	3	228.5156	505.7811	226.2176
	4	229.2292	505.0264	226.3685
	5	230.8061	505.2668	226.2794
	6	229.1044	505.5893	226.1105
	7	229.4713	505.0532	226.3775
	8	228.8252	505.2647	226.5558
	9	228.2853	505.0674	226.1972
	10	229.1981	505.4374	226.3370
Statistical error		229.1245±1.1620	505.3257±0.4150	226.3161±0.2138
**Experiment data2**	1	101.1739	120.6927	96.4365
	2	101.1811	120.1763	96.0172
	3	101.1949	120.5391	95.7914
	4	101.0158	120.1275	95.9908
	5	101.6481	120.0410	96.6969
	6	101.6621	120.5354	96.6614
	7	101.0856	120.0486	96.2066
	8	101.5261	120.5600	96.2366
	9	101.4354	120.8170	95.9727
	10	101.6277	120.8078	96.1094
Statistical error		101.2551±0.5277	120.4545±0.5196	96.2120±0.5091

### 3.4 Discussions

#### 3.4.1 Discussion on the influence of EMS parameters

**Fig 7:**
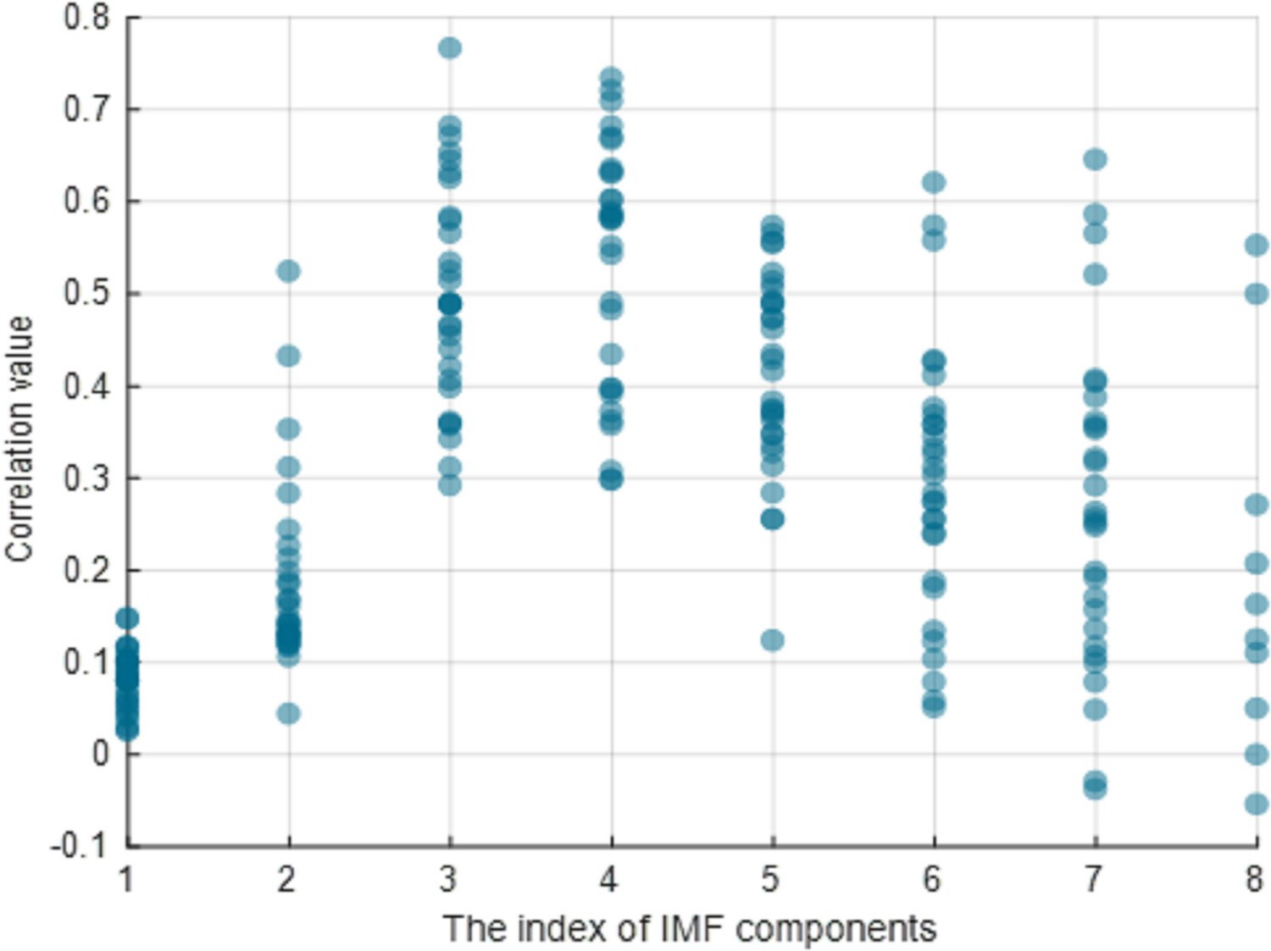
Results of the correlation between sets of IMFs and raw sEMG signals. The horizontal and vertical axes present the index of each IMF in a set of IMFs and the correlation value, respectively.

**Fig 8:**
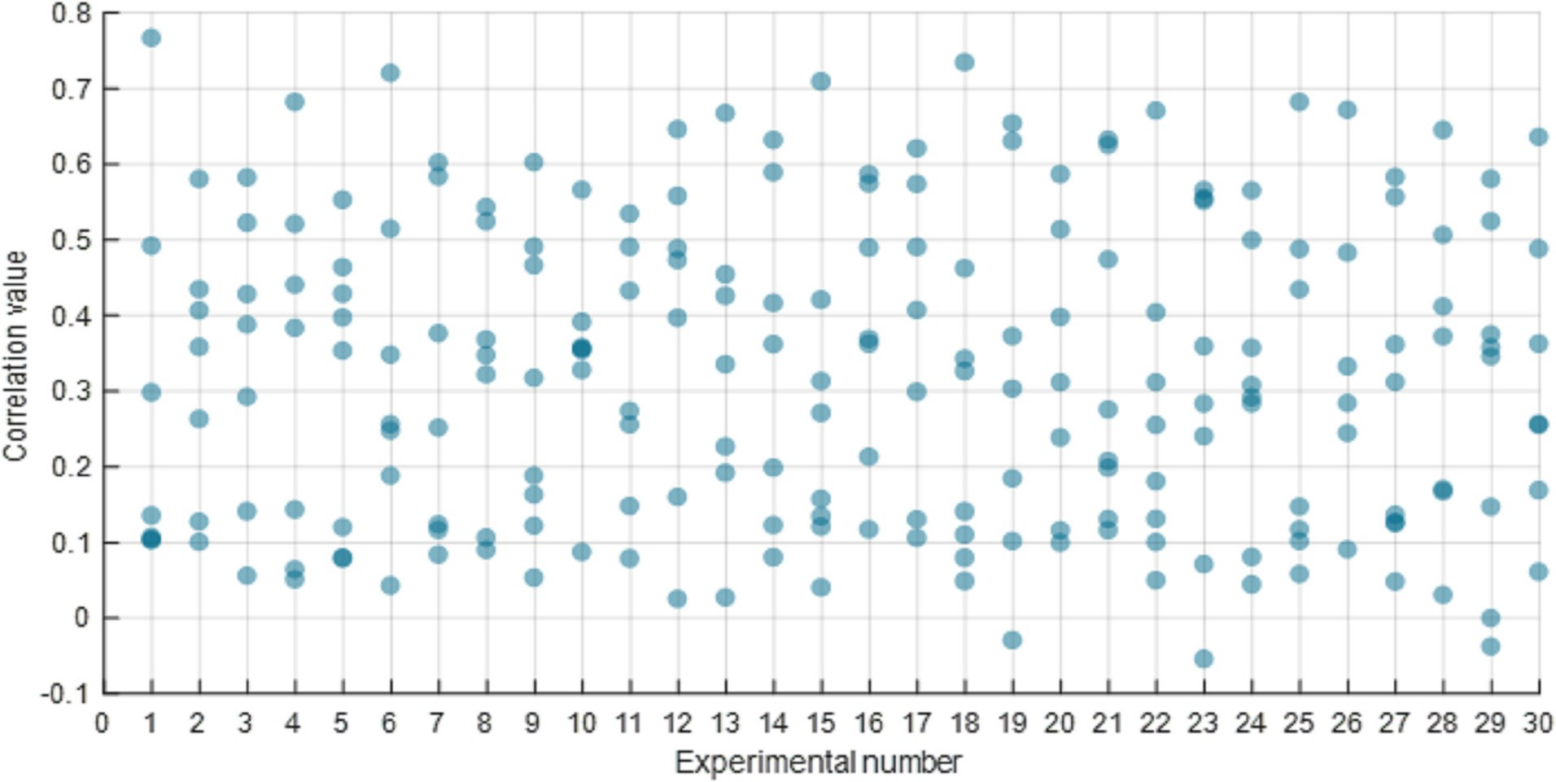
Result of the correlation between the sets of IMFs and raw sEMG signals. The horizontal and vertical axes present the serial number of experiment and the correlation value, respectively.

According to Section 2.1.3, before screening the IMFs using NMFSEMD, the upper and lower bounds of the correlation should be determined by comparing the preseted correlation threshold cr_*th*_ and the empirical maximum correlation cr_*e*_. The cr_*th*_ represents the boundary between the IMFs that should be discarded and the IMFs that should be denoised, and it has a small value which is usually less than 0.5. The cr_*e*_ represents the boundary between the IMFs, which should be denoised, and the IMFs, which can be directly used to construct the denoised sEMG signal, and it has a big value which is usually bigger than 0.5. To determine the values of both these parameters, we counted the correlation of 30 sets of IMFs, as shown in Figs 7 and 8.

After the EMD decomposition of raw sEMG signal, a set of IMFs with position indexes at {1, 2, 3, …} can be obtained. The correlation distribution of each index in a set of IMFs is shown in Fig 7. The correlation at the 1st index is less than 0.2 while those at the 2nd, 6th, 7th, and 8th indexes are distributed in a range of 0.0 to 0.4. Moreover, the correlation at the 3rd, 4th, and 5th indexes are distributed at a higher value in a range of 0.3 to 0.7. Therefore, we set the maximum of the correlation at indexes of 3rd, 4th, and 5th as the *cr*_*e*_, which limited the upper bound to a big value and ensured that the IMF whose correlation was greater than this value had a high correlation with the raw signal and contained most of the useful information about the raw signal. In the 30-group experiments, *cr*_e_ can be written as *cr*_*e*_ = 0.6125±0.0744. Fig 8 shows that the correlation distribution of IMFs in each experiment is more evenly distributed between 0.0 and 0.8. Therefore, in this study, the *cr*_*th*_, which can distinguish between the IMFs with small correlation (IMFs that need discarding) and the IMFs with medium correlation (IMFs that need denoising), is set in the range of 0.3 to 0.4.

### 3.4.2 Discussion on the influence of NMF parameters

In the abovementioned discussion, before using NMF to separate noise from sEMG signal, the rank *r* of matrix *W* and *H* should be preseted; where *r* also represents the number of muscle activation modes in a sEMG signal. Therefore, the performance of NMF method with different numbers of *r* can be discussed; and it also can be evaluated through the reconstruction rate (RSR) parameter, which is proposed in [40] and can be calculated using Eq (10). The larger the *RSR* value is, the more reasonable the setting of the number of activation modes and the more reliable the activation information obtained from *H* would be.

$$RSR=1-\frac{{\left\|V-WH\right\|}^{2}}{{\left\|V\right\|}^{2}}$$ (10)

where, *V*_*k* × *n*_ is the input matrix of NMF, and *W*_*k* × *r*_ and *H*_*r* × *n*_ are the output matrices of NMF.

When setting different numbers of activation modes, different matrices of *W* and *H* are obtained. Table 5 shows the *RSR* results of 6 groups of sEMG data with different numbers of *r*. Let us take Data 1 as an example. When *r* is 2, the *RSR* of *V* is 94.37%, which is large enough to represent muscle activity. When *r* is increased to 3, the *RSR* is 94.53%, which is an increase of < 0.5%. Therefore, continuing to increase the rank of matrix will not only bring more useful information but increase the noise-related information and subsequent amount of calculation, as shown in other groups of data in Table 5. Therefore, setting the number of muscle activation modes (*r*) to 2 in this study is suitable for sEMG signal.

**Table 5:** Calculated reconstruction rate of multiple groups of different sEMG signals under different rank values using NMF method.

*RSR*/%	The rank of *W* and *H*				
	1	2	3	4	5
Data 1	93.86	94.37	94.53	94.73	95.10
Data 2	99.23	99.23	99.22	99.27	99.27
Data 3	97.68	97.71	97.96	98.08	98.05
Data 4	98.33	98.34	98.53	98.72	98.68
Data 5	94.95	96.91	98.18	94.88	96.88
Data 6	97.64	97.79	97.87	97.95	98.02

## 4 Conclusions

In this study, an NMFSEMD algorithm is proposed to denoise the sEMG signals by analyzing the characteristics of non-stationarity and non-linearity of sEMG signals. First, the sEMG signal is decomposed into a set of IMFs, each of which contains different time scale characteristics. Then, the role of each IMF is distinguished in the denoised sEMG signal using the correlation between each IMF and the raw sEMG signal. Consequently, the screen operation of IMFs is performed to remove or reduce noise in each IMF. Finally, the denoised sEMG signal is obtained using the rule of reconstruction. By analyzing the results of the SNR and EP and the comparison of intuitive curves, it can be concluded that NMFSEMD can effectively filter out the noise of sEMG signals. For the muscle force prediction based on sEMG signals, the FOS algorithm in system identification is used to establish prediction model. The key point in establishing the model lies in setting of the form of candidate functions. The commonly used forms in sEMG applications are square, square-root, sigmoid activation functions, etc. Considering the input sEMG signal is the integral electromyogram value, a candidate function in the form of a derivative is added in this paper. In the experiment, when the input is the signal denoised using the NMFSEMD, the MSE value of FOS prediction model will be less than that of the LSSVM method. When the prediction model is FOS and the input data are the sEMG signals (with traditional preprocessing) and the sEMG signal denoised using the NMFSEMD, the MSE value of the latter will considerably be lower than that of the former. Comparing the predicted muscle force curve with the measured muscle strength curve, it can be concluded that the FOS prediction model uses the NMFSEMD-based denoised sEMG signal can achieve a better prediction result.

## Supporting information

S1 Dataset (ZIP)
